# Steady‐state cerebral blood flow and dynamic cerebral autoregulation during neck flexion and extension in seated healthy young adults

**DOI:** 10.14814/phy2.15622

**Published:** 2023-02-17

**Authors:** Tomokazu Kato, Toru Konishi, Takuya Kurazumi, Yojiro Ogawa, Ken‐ichi Iwasaki

**Affiliations:** ^1^ Department of Social Medicine, Division of Hygiene Nihon University School of Medicine Tokyo Japan; ^2^ Air Staff Office, Japan Air Self‐Defense Force Tokyo Japan; ^3^ Institute for Exercise and Environmental Medicine Texas Health Presbyterian Hospital Dallas Dallas Texas USA; ^4^ Department of Neurology University of Texas Southwestern Medical Center Dallas Texas USA

**Keywords:** cerebral circulation, dynamic cerebral autoregulation, head position, neck extension, neck flexion

## Abstract

Neck flexion and extension show differences in various physiological factors, such as sympathetic nerve activity and intracranial pressure (ICP). We hypothesized that differences would exist in steady‐state cerebral blood flow and dynamic cerebral autoregulation between neck flexion and extension in seated, healthy young adults. Fifteen healthy adults were studied in the sitting position. Data were collected during neck flexion and extension in random order for 6 min each on the same day. Arterial pressure at the heart level was measured using a cuff sphygmomanometer. Mean arterial pressure at the middle cerebral artery (MCA) level (MAP_MCA_) was calculated by subtracting the hydrostatic pressure difference between heart and MCA levels from mean arterial pressure at the heart level. Non‐invasive cerebral perfusion pressure (nCPP) was estimated as the MAP_MCA_ minus the non‐invasive ICP as determined from transcranial Doppler ultrasonography. Waveforms of arterial pressure in the finger and blood velocity in the MCA (MCAv) were obtained. Dynamic cerebral autoregulation was evaluated by transfer function analysis between these waveforms. The results showed that nCPP was significantly higher during neck flexion than during neck extension (*p* = 0.004). However, no significant differences were observed in mean MCAv (*p* = 0.752). Likewise, no significant differences were observed in any of the three indices of dynamic cerebral autoregulation in any frequency range. Although non‐invasively estimated cerebral perfusion pressure was significantly higher during neck flexion than during neck extension, no differences in steady‐state cerebral blood flow or dynamic cerebral autoregulation were evident between neck flexion and extension in seated healthy adults.

## Graphical abstract

The present study evaluated steady‐state cerebral blood flow and dynamic cerebral autoregulation during neck flexion and extension in seated healthy adults. The present results suggest that there are no major differences in cerebral circulation between neck flexion and extension despite the significant difference in the index of cerebral perfusion pressure in seated healthy individuals.

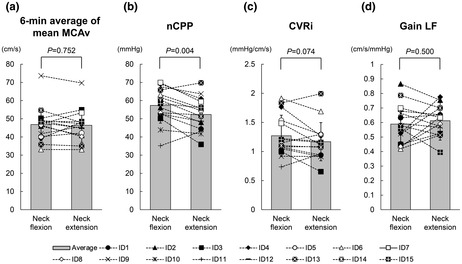

## 1 INTRODUCTION

Physiological factors influencing cerebral circulation may differ between head positions with neck flexion and extension in the upright body position. Confirming whether differences in cerebral circulation exist during neck flexion and extension may be beneficial to researchers. If cerebral circulation differs depending on head position with neck flexion and extension, researchers must pay attention to the head position of participants as a confounding factor when collecting data. In some cases, head position may be important for postlanding syndrome in astronauts (Williams et al., 2009), postflight data collection, and patients during rehabilitation.

Neck flexion and extension are associated with various physiological factors, such as changes in muscle sympathetic nerve activity, intracranial pressure (ICP), postural control, vestibular stimuli, neck muscle activity, and hydrostatic pressure. Neck flexion is well known to increase muscle sympathetic nerve activity (Carter & Ray, 2008; Essandoh et al., 1988; Hume & Ray, 1999), but neck extension does not affect muscle sympathetic nerve activity in a horizontal body position (Hume & Ray, 1999). Neck flexion has recently been shown to influence ICP as measured by lumbar puncture in a sitting position (Pedersen et al., 2021). Neck extension reportedly affects postural control in an upright position, whereas neck flexion has not been found to affect postural control (Kogler et al., 2000; Johnson & Van Emmerik R, 2012). In addition, the head moves back and forth across the direction of Earth's gravity during neck flexion and extension in the upright body position. Differences in the direction of head movement with respect to the Earth's gravity can produce differences in gravitational inputs to the otolith organs, neck muscle activity, and hydrostatic pressure in the brain, which can in turn influence the cerebral circulation via mechanisms such as the vestibulo‐sympathetic reflex, changes in arterial pressure, or ICP.

One possibility is that the above‐mentioned factors with neck flexion and extension in the upright body position may differently affect cerebral circulation. However, the differences between cerebral circulation under neck flexion and extension in the upright position have not previously been reported, although a few studies have investigated the effects of neck flexion and/or extension on cerebral circulation in a horizontal body position. Previous studies showed no effects of neck flexion and/or extension on steady‐state cerebral blood flow or dynamic cerebral autoregulation in a horizontal body position (Cooke et al., 2004; Wilson et al., 2003).

Here, we hypothesized that differences in cerebral circulation would exist between neck flexion and extension in an upright body position. To test this hypothesis, we evaluated steady‐state cerebral blood flow and dynamic cerebral autoregulation during neck flexion and extension in seated healthy adults.

## 2 METHODS

### 2.1 Participants

The study protocol was approved by the institutional review board of Nihon University School of Medicine (approval no. 30–6‐0) and was registered in the University Hospital Medical Information Network (UMIN) clinical trial registry (ID: UMIN000035059). All procedures adhered to the tenets of the Declaration of Helsinki. All participants provided written informed consent as well as a medical history regarding cardiovascular health and were screened based on a physical examination including electrocardiography and arterial pressure measurements. Exclusion criteria for health were as follows: failure to obtain blood velocity signals in the right middle cerebral artery (MCA) by transcranial Doppler ultrasonography (TCD); cardiovascular disease; autonomic disturbance; respiratory disease; current use of any medications; history of severe systemic disease; or allergy to the ultrasonic gel or polymer to be used in the study. Fifteen healthy adults (14 men, 1 woman) participated in the study. Mean (± standard deviation [SD]) age was 23 ± 2 years, mean height was 169 ± 4 cm, and mean weight was 64 ± 6 kg. All experiments were performed ≥2 h after a meal. Participants were asked to refrain from exercising heavily or consuming caffeinated or alcoholic beverages for at least 12 h before the experiments. All participants were familiarized with the measurement techniques and experimental conditions before the data collection experiments. We also confirmed that participants could smoothly flex and extend the neck 45° from the vertical plane and maintain that head position for 10 min without any complaints such as neck pain or difficulty breathing.

### 2.2 Procedure

The participant was seated on a chair in an environmentally controlled experimental room at an ambient temperature of 21–25°C. Both arms were placed on arm stands. A three‐lead electrocardiogram (ECG) and arterial oxygen saturation (SpO_2_) by pulse oximetry were monitored (Lifescope BSM‐3800; Nihon Kohden). An infrared carbon dioxide sensor that can obtain a capnogram via both the mouth and nose was applied to detect respiratory rate and end‐tidal carbon dioxide pressure (EtCO_2_) (OLG‐2800; Nihon Kohden). Arterial pressure waveforms were continually measured from the left middle finger using a volume clamping method with photoplethysmography as part of the feedback loop used to maintain clamping (Finometer MIDI; Finapres Medical Systems) and a height‐correction sensor was placed on the left arm at heart level (xiphoid process level). Arterial pressure at heart level was obtained by the oscillometric method with a cuff sphygmomanometer placed over the right brachial artery (Lifescope PT BSM‐6501; Nihon Kohden). Continuous waveforms of blood velocity in the MCA (MCAv) were obtained by TCD (EZ‐Dop; Compumedics Germany GmbH) at a depth of 50–60 mm using a 2‐MHz probe. The reproducibility of signals for MCAv from TCD is good when performed by an experienced sonographer and careful attention is paid to probe placement, as confirmed by the high intraclass correlation coefficient of approximately 0.9 (Brodie et al., 2009) and a small coefficient of variation (10%) in repeated baseline measurements (Iwasaki et al., 2007) of MCAv. The probe was positioned on the right temporal window and fixed at a constant angle that gave the highest MCAv and an appropriate Doppler signal. TCD monitoring with high reproducibility was achieved using a custom‐developed probe holder made of a polymer mold to fit the ear and facial bone structure of each individual participant. All TCD measurements were performed by the same experienced technician. Each waveform (ECG, capnography, arterial pressure in the finger, and MCAv) was recorded at a sampling rate of 1 kHz using commercial software (Notocord‐hem 3.3; Notocord) throughout the experiment.

#### 2.2.1 Protocols

**FIGURE 1:**
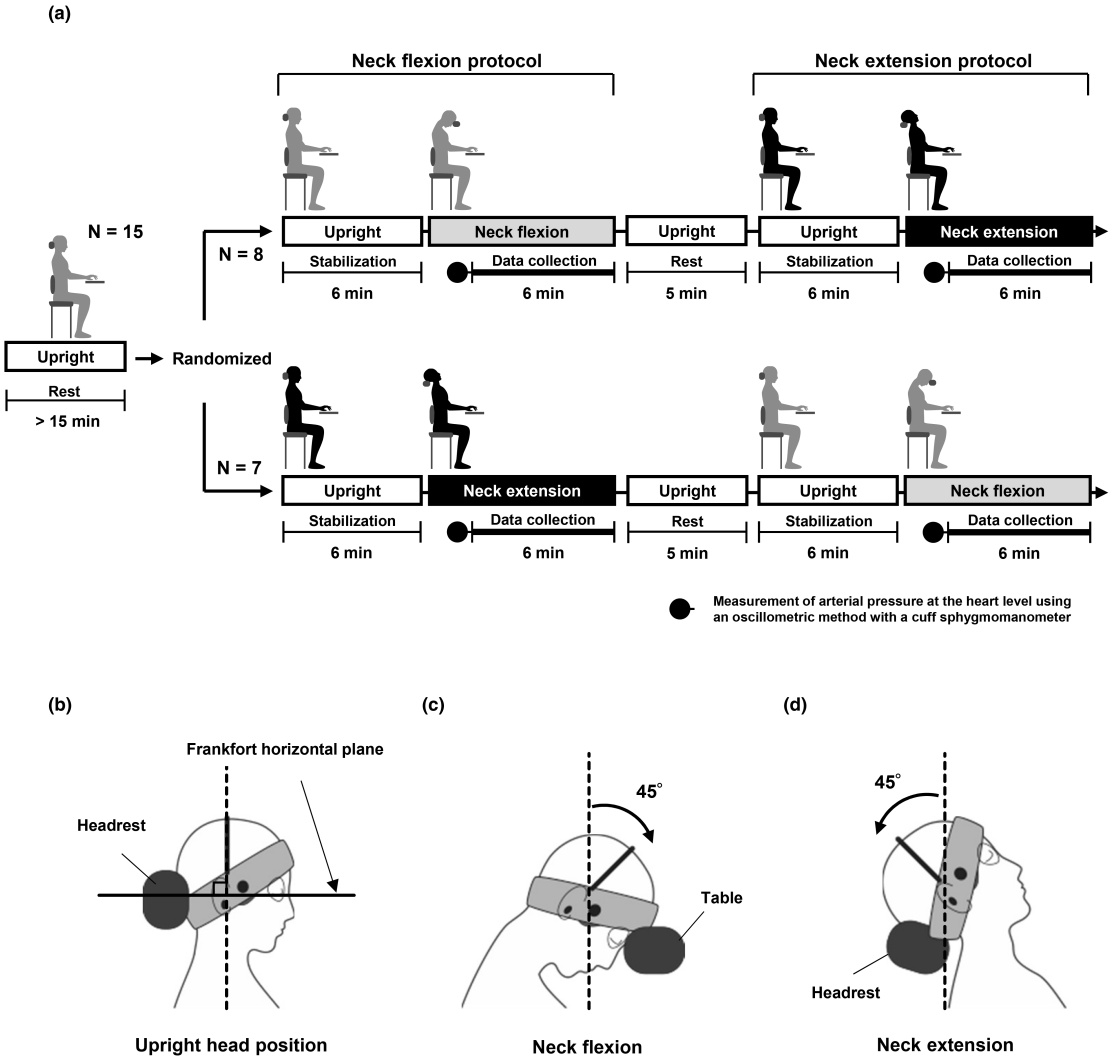
Experimental protocol (a). The present study comprised a neck flexion protocol and a neck extension protocol. After >15 min of quiet rest in a sitting position, these protocols were performed in random order on the same day. Of the 15 participants, eight participants started with the neck flexion protocol and seven participants started with the neck extension protocol. A 5‐min interval was provided between the two protocols. In each protocol, after stabilization of each measured waveform was confirmed for 6 min in an upright head position (0°, vertical), participants were asked to flex or extend their neck by 45°. After arterial pressure at heart level was measured by a cuff sphygmomanometer during neck flexion or extension, waveforms of arterial pressure in the finger and blood velocity in the middle cerebral artery were collected for 6 min. Schematic diagram showing each head position (b, upright head position; c, neck flexion; d, neck extension). To set the upright head position for the participant, the Frankfort horizontal plane was aligned with the horizontal plane (b). The back of the head was supported by a headrest in the upright position. The participants were asked to flex or extend their neck 45° forward or backward and to maintain that position (c, d). The forehead was supported by a table during neck flexion and the back of the head was supported by a headrest during neck extension (c, d).

Graphical representations of the experimental protocol are shown in Figure 1a. The present study comprised a neck flexion protocol and a neck extension protocol. After >15 min of quiet rest in a sitting position, these protocols were performed in random order on the same day. Of the 15 participants, eight participants started with the neck flexion protocol and seven participants started with the neck extension protocol. A 5‐min interval was provided between the two protocols. After stabilization of each measured waveform was confirmed for 6 min in the upright head position (0°, vertical) (Figure 1b), the participants were asked to flex their neck 45° forward from the vertical plane (Figure 1c) or extend their neck 45° backward (Figure 1d), and to maintain that head position. The change in head position from upright to neck flexion or extension was promptly performed voluntarily without any external influence. Following measurements of intermittent arterial pressure at the heart level using a cuff sphygmomanometer during neck flexion or extension, data for neck flexion or extension were collected for 6 min. The participant was blindfolded and instructed to keep their eyes closed to avoid visual inputs.

#### 2.2.2 Steady‐state hemodynamics and respiratory conditions

Mean arterial pressure at the heart level (MAP_Heart_) was measured by the cuff sphygmomanometer during neck flexion or extension (Figure 1a). To determine the mean arterial pressure at the MCA level (MAP_MCA_), hydrostatic pressure difference between the heart level and external acoustic meatus level was subtracted from the MAP_Heart_. Distances from the heart level to the external acoustic meatus level (Distance_Heart‐MCA_) were measured for each head position. The external acoustic meatus level was applied as the MCA level in the present study. The hydrostatic pressure difference (mm Hg) was calculated as follows:

$\begin{array}{c} \text{hydrostatic pressure difference}\;\left(\mathrm{mm}\;\mathrm{Hg}\right)= \end{array}$ $\begin{array}{c} {\text{Distance}}_{\text{Heart}-\mathrm{MCA}}\;\left(\mathrm{cm}\right)\times 1.06/13.6\times 10 \end{array}$.

This is based on the assumption that the specific gravity of mercury at 37°C (density: 13,500 kg/m^3^) with reference to water at 37°C (density: 993 kg/m^3^) is 13.6, and the specific gravity of whole blood at 37°C with reference to water at 37°C is 1.06 (Trudnowski & Rico, 1974).

Six‐minute averages of heart rate (HR), mean MCAv, SpO_2_, respiratory rate, and EtCO_2_ were obtained for each data collection period as steady‐state parameters.

#### 2.2.3 Non‐invasive ICP, cerebral perfusion pressure, and cerebrovascular resistance index

Non‐invasive ICP (nICP) was calculated using the plugin “nICP Plugin” software (Klinkum Chemnitz GmbH) for ICM+ version 8.1 (Cambridge Enterprise) based on the mathematical model described by Schmidt et al. (1997). This model is based on a concept of system analysis in which output signals (i.e., ICP) result from a systemic response to input signals (i.e., arterial pressure) (Schmidt et al., 1997). Analyses for nICP were performed as described in previous studies (Kato et al., 2022). Briefly, waveforms for continuous arterial pressure at the heart level and MCAv recorded at 1 kHz were resampled at 100 Hz using Notocord‐Hem 3.3 software (Notocord) for this analysis. Waveforms for arterial pressure at the MCA level were calculated using arterial pressure at the heart level and hydrostatic pressure difference in each head position as mentioned above. Calculations for nICP were performed for every 10‐s window, and values of 36 windows (6 min) were averaged.

Non‐invasive cerebral perfusion pressure (nCPP) was also calculated by subtracting nICP from MAP_MCA_. Cerebrovascular resistance index (CVRi) was calculated as nCPP divided by the 6‐min average of MCAv (Kato et al., 2022).

#### 2.2.4 Spectral and transfer function analyses

**FIGURE 2:**
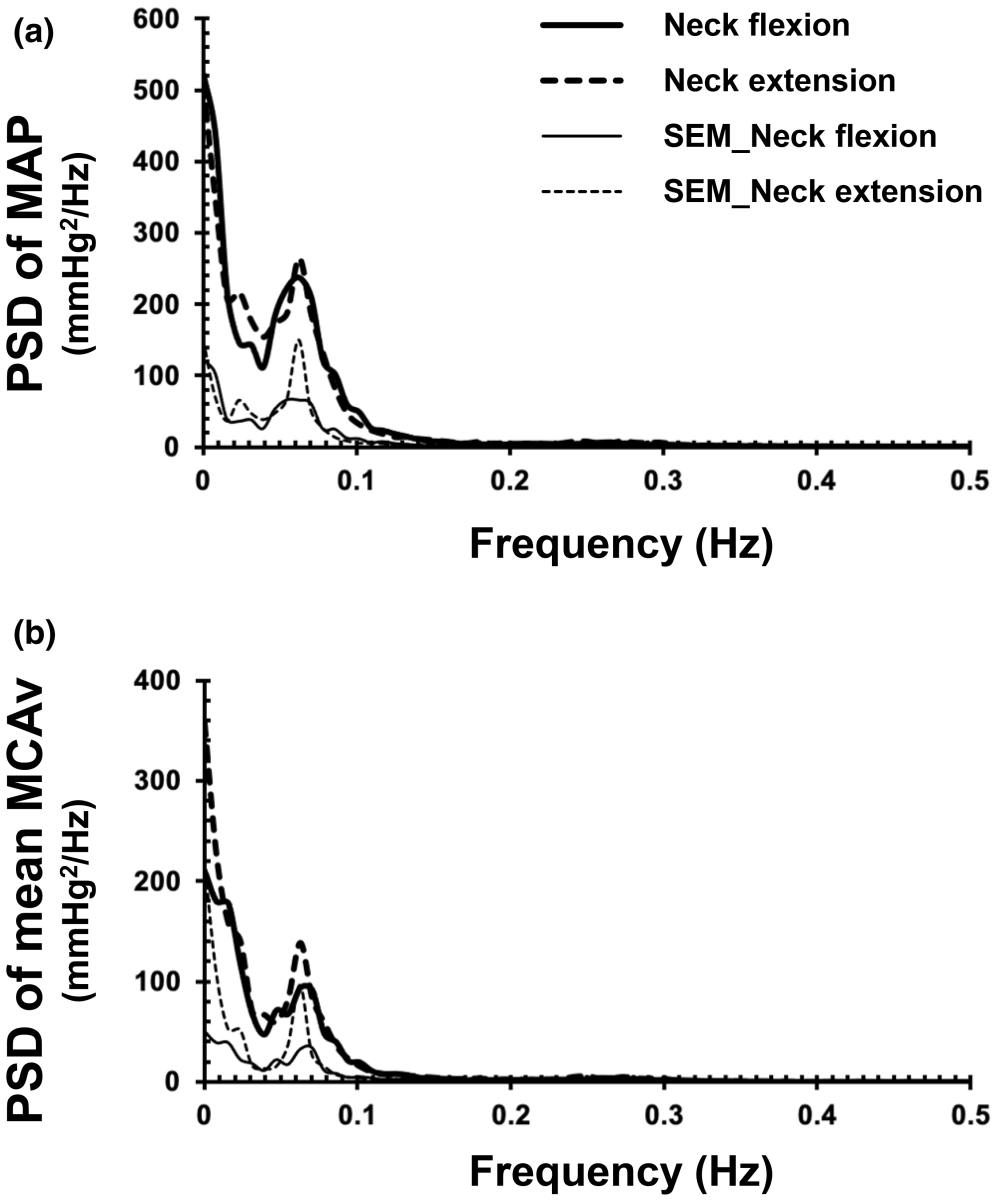
Group‐averaged power spectral density (PSD) of mean arterial pressure (MAP) (a) and mean blood velocity in the middle cerebral artery (MCAv) (b) during neck flexion and extension (*N* = 15). SEM, standard error of the mean.

**FIGURE 3:**
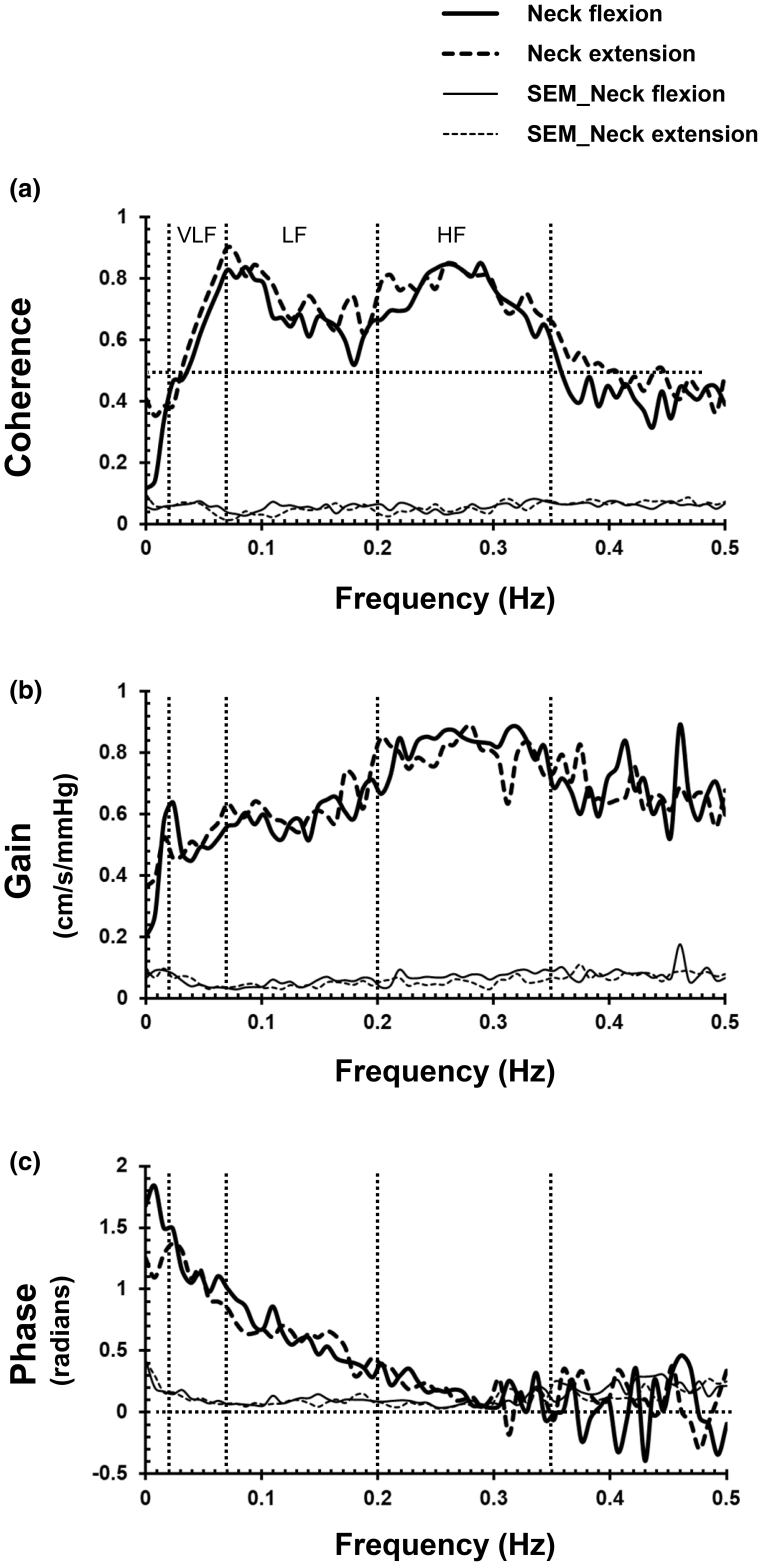
Frequency responses of transfer function indices between mean arterial pressure (MAP) and mean blood velocity in the middle cerebral artery (MCAv) variabilities during neck flexion and extension (*N* = 15). Coherence, coherence function (a); Gain, transfer function gain between MAP and mean MCAv variabilities (b); Phase, phase relationship between MAP and mean MCAv variabilities (c); VLF, very low‐frequency range (0.02–0.07 Hz); LF, low‐frequency range (0.07–0.20 Hz); HF, high‐frequency range (0.20–0.35 Hz); SEM, standard error of the mean.

Beat‐to‐beat values of mean arterial pressure (MAP) and mean MCAv were obtained by integrating signals within each cardiac cycle from the waveforms of arterial pressure in the finger and MCAv using PC‐based commercial software (Notocord‐hem 3.3). Beat‐to‐beat data were then linearly interpolated and resampled at 4 Hz for spectral and transfer function analyses. Time series of data was first subtracted by the 6‐min average for variabilities. Fast Fourier transform and transfer function analyses were performed using a Hanning window on 512‐point segments with 50% overlap. This process resulted in five segments of data recordings over 6 min from which to assess the dynamic pressure‐flow relationship (Panerai et al., 2023). We analyzed these data using DADiSP software (DSP Development). The spectral power of MAP variability and mean MCAv variability (Figure 2), and transfer function gain, phase, and coherence were calculated in the very low frequency (0.02–0.07 Hz), low frequency (0.07–0.20 Hz), and high frequency (0.20–0.35 Hz) ranges (Figure 3). These ranges were specifically based on the frequency‐dependent property of dynamic cerebral autoregulation as previously proposed by transfer function analysis (Zhang et al., 2002). Coherence ranging between 0 and 1 reflects a linear relationship between MAP and mean MCAv variabilities. Transfer function gain reflects the capability of the distal cerebral arterioles to suppress transmission from MAP oscillation to mean MCAv fluctuation. Transfer function gain is represented as the absolute value and the relative value normalized to MAP and mean MCAv in accordance with the recommendations of the white paper by Panerai et al. (2023). The phase is the temporal relationship between MAP and mean MCAv variabilities. A positive phase value indicates that changes in mean MCAv show the phase leads to changes in MAP, and zero indicates that changes in mean MCAv are synchronized with changes in MAP without effective dynamic cerebral autoregulation against rapid changes in MAP. To avoid the occurrence of phase “wrap‐around,” negative values of phase detected for frequencies <0.1 Hz were removed (Panerai et al., 2023).

#### 2.2.5 Statistical analysis

Data are presented as mean ± SD. All statistical analyses were performed using SygmaPlot software (version 14.5; Systat Software). The normality of data distributions was confirmed using the Kolmogorov–Smirnov test. A paired *t* test was used for variables showing a normal distribution, and the Wilcoxon signed‐rank test was used for variables with a non‐normal distribution. Values of *p* < 0.05 were considered to indicate statistically significant differences.

## 3 RESULTS

**FIGURE 4:**
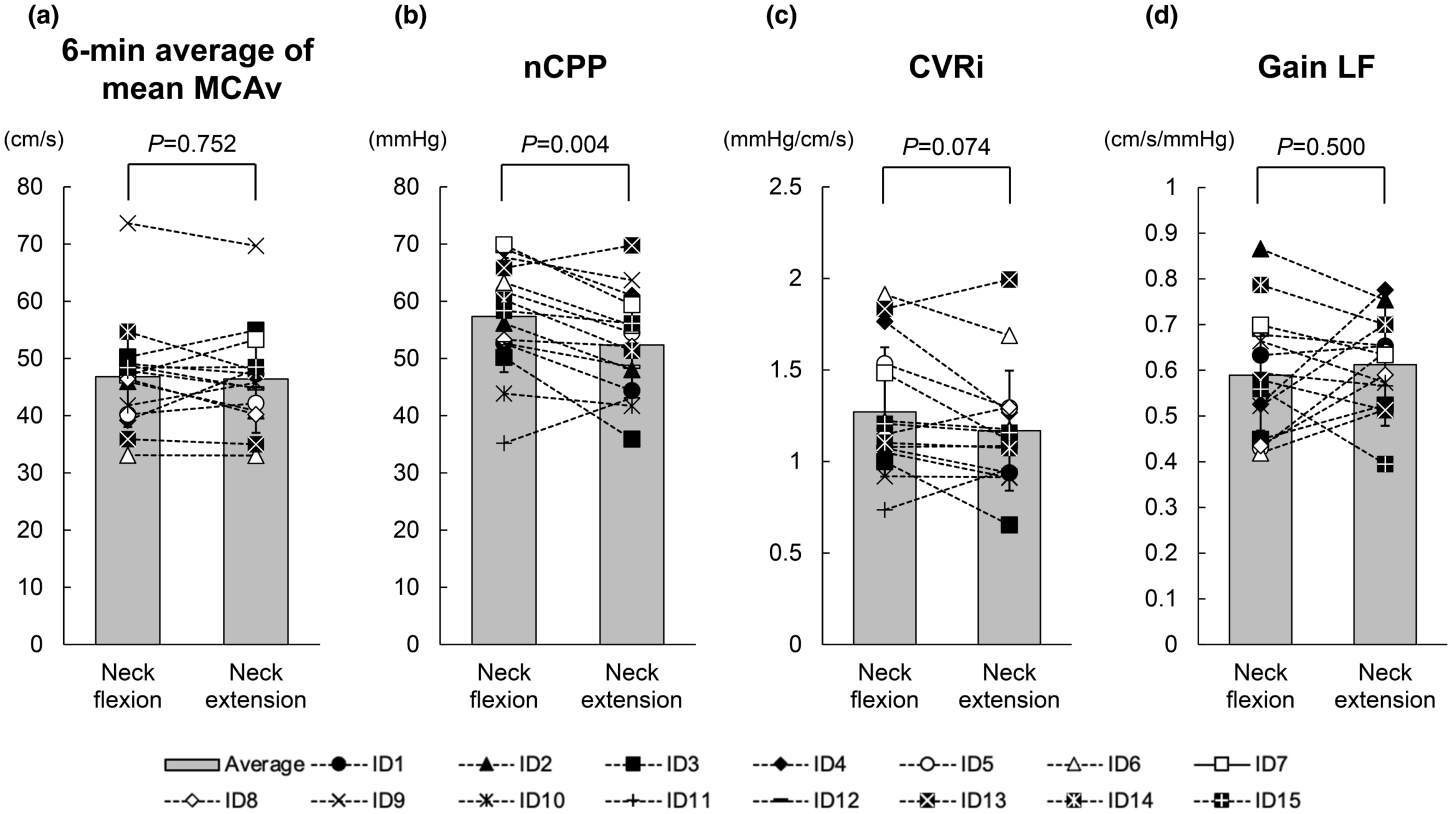
Group averages and individual changes in 6‐min average of mean blood velocity in the middle cerebral artery (MCAv) (a), non‐invasive cerebral perfusion pressure (nCPP) (b), cerebrovascular resistance index (CVRi) (c), and transfer function gain in the low‐frequency range (Gain LF) (d) during neck flexion and extension. A paired *t*‐test was used to determine significant differences between neck flexion and extension. Gray bars with error bars represent mean values and SDs for the 15 participants.

Table 1 shows group averages for steady‐state hemodynamics and respiratory conditions in each head position. MAP_Heart_ during neck flexion tended to be higher than during neck extension, but this difference was not significant (*p* = 0.062). MAP_MCA_ was significantly higher during neck flexion than during neck extension (*p* = 0.008). No significant difference in the 6‐min average of mean MCAv was evident between neck flexion and extension (*p* = 0.752) (Figure 4a). No significant difference in nICP was also evident between neck flexion and extension (*p* = 0.524). Values for nCPP were significantly higher during neck flexion than during neck extension (*p* = 0.004) (Figure 4b). CVRi tended to be higher during neck flexion than during neck extension, but this difference was not significant (*p* = 0.074) (Figure 4c). The 6‐min average of EtCO_2_ was significantly higher during neck extension than during neck flexion (*p* = 0.010). No significant differences in 6‐min averages of HR, respiratory rate, or SpO_2_ were noted between neck flexion and extension. The hydrostatic pressure difference between heart and MCA levels was significantly smaller during neck flexion than during neck extension (*p* < 0.001).

**TABLE 1:** Steady‐state hemodynamics and respiratory condition.

	Neck flexion	Neck extension	*p*‐value	
HR (beats/min)	71 ± 10	72 ± 11	0.690	(T)
MAP_Heart_ (mm Hg)	86 ± 9	83 ± 8	0.062	(T)
MAP_MCA_ (mm Hg)	64 ± 9	59 ± 8**	0.008	(T)
Mean MCAv (cm/s)	46 ± 9	46 ± 8	0.752	(T)
nICP (mm Hg)	6.6 ± 2.3	7.0 ± 2.8	0.524	(W)
nCPP (mm Hg)	57 ± 9	52 ± 9**	0.004	(T)
CVRi (mmHg/cm/s)	1.27 ± 0.35	1.16 ± 0.32	0.074	(T)
Resp‐R (breath/min)	16 ± 1	16 ± 2	0.859	(T)
SpO_2_ (%)	96 ± 0	96 ± 0	0.871	(T)
EtCO_2_ (mm Hg)	35 ± 2	36 ± 1*	0.010	(T)
Hydrostatic pressure difference (mm Hg)	22 ± 0	23 ± 1***	<0.001	(W)

*Note*: Values are means ± SD. *p*‐value are expresses as (T) *t*‐test or (W) Wilcoxon signed‐rank test.

Abbreviations: CVRi, cerebrovascular resistance index; EtCO_2_, end‐tidal carbon dioxide pressure; HR, heart rate; Hydrostatic pressure difference, hydrostatic pressure difference between the heart and the middle cerebral artery; MAP_Heart_, mean arterial pressure at the heart level; MAP_MCA_, mean arterial pressure at the middle cerebral artery level; Mean MCAv, mean blood velocity in the middle cerebral artery; nCPP, non‐invasive cerebral perfusion pressure; nICP, non‐invasive intracranial pressure; Resp‐R, respiratory rate; SpO_2_, arterial oxygen saturation.

* *p* < 0.05 (vs. Neck flexion)

** *p* < 0.01 (vs. Neck flexion)

*** *p* < 0.001 (vs. Neck flexion).

Table 2 shows the results of spectral and transfer function analyses, and Figure 2 shows the group‐averaged power spectral density of MAP and mean MCAv variabilities. No significant differences were observed in the spectral power of MAP variability or mean MCAv variability in any of the frequency ranges between neck flexion and extension. Figure 3 shows the frequency responses of transfer function indices between MAP and mean MCAv variabilities, and Figure 4d shows group averages and individual changes in transfer function gain in the low‐frequency range. No significant differences in coherence or transfer function gain or phase were identified in any of the frequency ranges between neck flexion and extension. Frequency responses of all three indices of transfer function analysis between MAP and mean MCAv variabilities were almost identical between neck flexion and extension (Figure 3).

**TABLE 2:** Spectral and trasfer function analysis.

	Neck flexion	Neck extension	*p*‐value	
VLF				
VLF‐MAPv (mmHg^2^)	7.88 ± 7.57	8.26 ± 9.76	0.803	(T)
VLF‐MCAvv (cm^2^/s^2^)	3.18 ± 3.15	3.76 ± 5.15	0.481	(T)
Coherence‐VLF	0.59 ± 0.16	0.62 ± 0.14	0.413	(T)
Gain‐VLF (cm/s/mmHg)	0.51 ± 0.11	0.50 ± 0.15	0.916	(T)
NGain‐VLF (%/%)	0.95 ± 0.30	0.92 ± 0.24	0.566	(T)
Phase‐VLF (radians)	1.14 ± 0.26	1.12 ± 0.23	0.807	(T)
LF				
LF‐MAPv (mmHg^2^)	3.92 ± 3.07	3.45 ± 2.19	0.309	(T)
LF‐MCAvv (cm^2^/s^2^)	1.62 ± 1.20	1.62 ± 1.18	0.997	(T)
Coherence‐LF	0.68 ± 0.10	0.74 ± 0.11	0.066	(T)
Gain‐LF (cm/s/mmHg)	0.58 ± 0.13	0.61 ± 0.10	0.500	(T)
NGain‐LF (%/%)	1.09 ± 0.25	1.14 ± 0.24	0.429	(T)
Phase‐LF (radians)	0.62 ± 0.21	0.61 ± 0.18	0.771	(T)
HF				
HF‐MAPv (mm Hg^2^)	0.48 ± 0.36	0.61 ± 0.69	0.188	(W)
HF‐MCAvv (cm^2^/s^2^)	0.34 ± 0.19	0.44 ± 0.40	0.639	(W)
Coherence‐HF	0.74 ± 0.09	0.76 ± 0.07	0.374	(T)
Gain‐HF (cm/s/mm Hg)	0.81 ± 0.18	0.79 ± 0.11	0.491	(T)
NGain‐HF (%/%)	1.48 ± 0.20	1.46 ± 0.16	0.740	(T)
Phase‐HF (radians)	0.17 ± 0.14	0.17 ± 0.09	0.905	(T)

*Note*: Values are means ± SD. *p*‐value are expresses as (T) *t*‐test or (W) Wilcoxon signed‐rank test.

Abbreviations: HF, high‐frequency range (0.20–0.35 Hz); LF, low‐frequency range (0.07–0.20 Hz); MAPv, mean arterial pressure at the heart level variability; MCAvv, mean blood velocity in the middle cerebral artery variability; Gain, transfer function gain; NGain, normalized transfer function gain; VLF, very low‐frequency range (0.02–0.07 Hz).

## 4 DISCUSSION

Three major findings were obtained in the present study. First, nCPP during neck flexion was higher than that during neck extension in the upright sitting body position. Second, no significant differences in the 6‐min average of mean MCAv were apparent between neck flexion and extension in the upright sitting position. Third, no significant differences were identified in any indices of dynamic cerebral autoregulation between neck flexion and extension in the upright sitting position. Counter to our hypothesis, the present results suggest that steady‐state cerebral blood flow and dynamic cerebral autoregulation may not show any significant differences between neck flexion and extension in the upright body position, despite the higher non‐invasively estimated cerebral perfusion pressure during neck flexion than during neck extension.

The present results showed that nCPP was significantly higher during neck flexion than during neck extension, suggesting higher cerebral perfusion pressure during neck flexion than during neck extension in the upright sitting position. The present study applied equivalent tilting angles (45°) from the vertical plane for head positions during neck flexion and extension, resulting in a lower head position during neck flexion than during neck extension. The differences in height of the MCA between neck flexion and extension may be the primary reason for differences in nCPP as calculated by “MAP_Heart_ – hydrostatic pressure difference – nICP”. In addition, MAP_Heart_ tended to be higher during neck flexion than during neck extension in the present study. A previous study showed that muscle sympathetic nerve activity increased during neck flexion, but not during neck extension in a prone position (Hume & Ray, 1999). Differential response in muscle sympathetic nerve activity between neck flexion and extension might also occur in the upright sitting position, which could have contributed to the differences in nCPP observed in the present study.

The present results showed that the 6‐min average of mean MCAv did not differ between neck flexion and extension in the sitting position. This result suggests a lack of difference in steady‐state cerebral blood flow between neck flexion and extension in the upright body position, consistent with a previous study conducted in the horizontal body position (Wilson et al. 2003). In general, steady‐state cerebral blood flow is maintained relatively constant by adjusting arteriolar resistance in the brain within a limited range of cerebral perfusion pressure. The present study showed that CVRi tended to be higher during neck flexion than during neck extension. These results for CVRi support the interpretation that arteriolar resistance adjusts to maintain cerebral blood flow at a near‐constant level under changes to cerebral perfusion pressure arising from alterations in head position. Although arguments have recently been put forward regarding the range and degree of variation among individuals (Drummond, 2019), the difference in cerebral perfusion pressure between neck flexion and extension in the present study was likely within the range where adjustment of arteriolar resistance is effective. In addition, no significant differences in any transfer function indices were observed for any frequency ranges between neck flexion and extension in the present study. These results are again consistent with a previous study conducted with subjects in a horizontal body position (Cooke et al., 2004). Furthermore, the frequency responses of all three indices of transfer function analysis were almost identical between neck flexion and extension (Figure 3). Thus, the present results from 6‐min averages of mean MCAv and dynamic cerebral autoregulation indices together suggest a lack of major differences in cerebral circulation between neck flexion and extension in the upright body position.

One key factor that can influence cerebral circulation is ICP. A previous study showed that cerebrospinal fluid pressure as measured by lumbar puncture in a sitting position was higher during neck flexion than with a straight neck (Pedersen et al., 2021). ICP could potentially change depending on head position due to changes in height of the brain and/or cerebral venous drainage caused by mechanical stress on the internal jugular veins. However, the present study found no significant differences in nICP between neck flexion and extension.

The present study showed that EtCO_2_ was slightly (~1 mm Hg) but significantly higher during neck extension than during neck flexion, consistent with findings from a previous study (Wilson et al., 2003). Other investigations have reported that tidal volume decreases (Kim et al., 2017) and anatomical dead space increases (Nunn et al., 1959) during neck extension compared to the neutral head position. Decreases in tidal volume and increases in anatomical dead space would have resulted in an increased partial pressure of arterial CO_2_ (PaCO_2_) and higher EtCO_2_ during neck extension in the present study. Although increases in PaCO_2_ have been shown to increase steady‐state cerebral blood flow and to weaken dynamic cerebral autoregulation (Panerai et al., 1999; Zhang et al., 1998), the slight difference in EtCO_2_ (~1 mm Hg) between neck flexion and extension in the present study was unlikely to have any obvious effects on steady‐state MCAv or indices of dynamic cerebral autoregulation.

### 4.1 Limitations

Some limitations in the present study must be considered when attempting to interpret the findings. First, the evaluation of cerebral blood flow by TCD may have been affected by changes in MCA diameter, the angle of isolation, the position of the isolation window, and/or the position of the MCA in the brain during the neck flection and extension. However, the frequency response of phase, which may be minimally affected by changes in these factors, was almost identical between neck flexion and extension in the present study. Second, heterogeneity in vasomotor control along the arterial tree may disrupt the concept of transfer function analysis using velocity in the MCA as a conduit artery to indicate the capability of arterioles in some situations (e.g., changes in compliance). Third, the present study evaluated only the right MCA under TCD. Previous studies have shown hemispheric differences in the response of cerebral blood flow during mental tasks or dominant arm movements (Barnes et al., 2022; Silvestrini et al., 1994). Hemispheric differences in cerebral circulation might thus have been missed in the present study, although neck flexion and extension are symmetrical body motions and we tried to avoid visual, auditory, and psychological inputs during data collection as much as possible. Fourth, the present study did not directly measure ICP. The software used for estimating ICP in seated healthy participants was developed based on data from neurosurgical patients (Schmidt et al., 1997). We acknowledge the possibility that differences in ICP between neck flexion and extension might not have been detected due to inaccuracies in ICP estimation in the present study. Fifth, we used absolute angles (45°) for defining neck flexion and extension. Efforts to maintain a head position at 45° may differ between neck flexion and extension due to differences in this as a percentage of the total range of motion. Finally, we did not attempt to balance the sex ratio of participants during recruitment and only one woman applied to participate in this study. Thus, the findings of the present study may apply mainly to healthy men rather than being representative human data. Steady‐state cerebral blood flow tends to be faster in females than in males (Vavilala et al., 2005) and has also been shown to increase during the late follicular phase of the menstrual cycle (Peltonen et al., 2016). Sex differences in cerebral circulation and the timing of experimental measurements in female subjects could thus potentially produce different results. To clarify possible sex differences in the present findings, especially for MCAv and indices of dynamic cerebral autoregulation, future studies with enough women participants and data collection times controlling for the menstrual cycle are needed (Clayton, 2018).

## 5 CONCLUSION

The present study evaluated steady‐state cerebral blood flow and dynamic cerebral autoregulation during neck flexion and extension in seated healthy adults. Contrary to our hypothesis, the present study found no significant differences in the 6‐min average of mean MCAv or any indices of dynamic cerebral autoregulation between neck flexion and extension, despite significantly higher nCPP during neck flexion than during neck extension. These results suggest that there are no major differences in cerebral circulation between neck flexion and extension despite the significant difference in the index of cerebral perfusion pressure in seated healthy individuals.

## AUTHOR CONTRIBUTIONS

TKa helped with the design of the study, acquisition, analysis and interpretation of data, and drafting of the manuscript. TKo, TKu, and YO helped with the acquisition and interpretation of data and revision of the manuscript. KI helped with the conception and design of the study, acquisition, analysis, and interpretation of data, and revision of the manuscript. All authors reviewed the final manuscript.

## FUNDING INFORMATION

This study was supported by the Ministry of Education, Culture, Sports, Science, and Technology KAKENHI Grant Number JP15H05939, as part of “Living in Space (Grant‐in‐Aid for Scientific Research on Innovative Areas [2015‐2019])”, and by JSPS KAKENHI grant number JP20K06844 for additional data analysis and editing of the manuscript.

## CONFLICT OF INTEREST STATEMENT

The authors declare that no conflicts of interest exist.

## ETHICS APPROVAL AND CONSENT TO PARTICIPATE

The study protocol was approved by the Institutional Review Board of Nihon University School of Medicine (approval no. 30‐6‐0). Written informed consent and medical histories were obtained from all participants before the study began.
